# TabZIP74 Acts as a Positive Regulator in Wheat Stripe Rust Resistance and Involves Root Development by mRNA Splicing

**DOI:** 10.3389/fpls.2019.01551

**Published:** 2019-11-27

**Authors:** Fengtao Wang, Ruiming Lin, Yuanyuan Li, Pei Wang, Jing Feng, Wanquan Chen, Shichang Xu

**Affiliations:** ^1^State Key Laboratory for Biology of Plant Diseases and Insect Pests, Institute of Plant Protection, Chinese Academy of Agricultural Sciences, Beijing, China; ^2^China Agricultural University, College of Plant Protection, Beijing, China

**Keywords:** common wheat, Puccinia striiformis f. sp. tritici, bZIP transcription factor, endoplasmic reticulum stress, mRNA splicing, disease resistance

## Abstract

Basic leucine zipper (bZIP) membrane-bound transcription factors (MTFs) play important roles in regulating plant growth and development, abiotic stress responses, and disease resistance. Most bZIP MTFs are key components of signaling pathways in endoplasmic reticulum (ER) stress responses. In this study, a full-length cDNA sequence encoding bZIP MTF, designated *TabZIP74*, was isolated from a cDNA library of wheat near-isogenic lines of Taichung29*6/*Yr10* inoculated with an incompatible race CYR32 of *Puccinia striiformis* f. sp. *tritici* (*Pst*). Phylogenic analysis showed that *TabZIP74* is highly homologous to *ZmbZIP60* in maize and *OsbZIP74* in rice. The mRNA of *TabZIP74* was predicted to form a secondary structure with two kissing hairpin loops that could be spliced, causing an open reading frame shift immediately before the hydrophobic region to produce a new TabZIP74 protein without the transmembrane domain. *Pst* infection and the abiotic polyethylene glycol (PEG) and abscisic acid (ABA) treatments lead to *TabZIP74* mRNA splicing in wheat seedling leaves, while both spliced and unspliced forms in roots were detected. In the confocal microscopic examination, TabZIP74 is mobilized in the nucleus from the membrane of tobacco epidermal cells in response to wounding. Knocking down *TabZIP74* with barley stripe mosaic virus-induced gene silencing (BSMV-VIGS) enhanced wheat seedling susceptibility to stripe rust and decreased drought tolerance and lateral roots of silenced plants. These findings demonstrate that *TabZIP74* mRNA is induced to splice when stressed by biotic and abiotic factors, acts as a critically positive regulator for wheat stripe rust resistance and drought tolerance, and is necessary for lateral root development.

## Introduction

Plant pathogens, including fungi, viruses, bacteria, oomycetes, and nematodes, cause severe yield losses in crop production. To defend themselves against disease, plants have evolved different defense mechanisms against attackers. The first layer of defense is activated by athogen-associated molecular patterns (PAMPs) or microbe-associated molecular patterns (MAMPs). This kind of defense is referred to as PAMP triggered immunity (PTI) or MAMP triggered immunity (MTI) and defend the plant against non-specialized pathogens. This type of defense is perceived by plasma membrane receptors, which rely on protein maturation and endoplasmic reticulum (ER) protein folding quality control (Li et al., 2009; Lu et al., 2009; Nekrasov et al., 2009; Saijo et al., 2009) and the secretion of anti-microbial proteins to the apoplast (van Loon et al., 2006).

Secretory proteins are synthesized and folded in ERs, and proper folding is needed to transport the proteins to their final destinations. Perturbations of this folding process result in ER stress, which is critical for rapid and effective basal immune responses of plant hosts (Wang et al., 2005). Regulation of ER capacity is important for immune signaling (Korner et al., 2015). The ER stress triggers cytoprotective signaling pathways, titled the unfold protein response (UPR), this signaling pathway restores and maintains ER homeostasis. There is growing recognition that ER stress responses are involved in normal plant development (Deng et al., 2016; Kim et al., 2018; Bao et al., 2019); for example, ER stress is involved in the cells synthesizing and secreting materials comprising the pollen coat (Deng et al., 2016).

There are two arms of the ER stress signaling pathway in plants, one of which encompass membrane-associated transcription factors (MTFs) through proteolytic cleavage, such as the MTFs of AtbZIP17 (Liu et al., 2007b), AtbZIP28 (Liu et al., 2007a; Tajima et al., 2008), ZmbZIP17 (Yang et al., 2013), and OsbZIP39 (Takahashi et al., 2012), which are mobilized from ERs to golgi apparatus in plant cell where they are released by site 1 and site 2 proteases (S1P and S2P) (Liu et al., 2007a; Sun et al., 2015). The other ER stress signaling pathway functions through inositol requiring enzyme 1 (IRE1) and the splicing of target RNA, such as *bZIP60*, which encodes an MTF of basic leucine zipper (bZIP) (Liu and Howell, 2010; Howell, 2013). Splicing of target mRNA lead a frame shift of ORF and produces a new protein without a transmembrane domain (TMD) in its C-terminal. Many MTF genes, such as *XBP1* in yeast (Yoshida et al., 2001), *AtbZIP60* in Arabidopsis (Deng et al., 2011), *OsbZIP50/OsbZIP74* in rice (Hayashi et al., 2012), and *ZmbZIP60* in maize (Li et al., 2012) are activated by the IRE1 depended mRNA splicing pathway.

The bZIP TF family is one of the largest families in plants, with the most diverse biological functions (Jakoby et al., 2002); several of its members, including *AtbZIP60*, *NtbZIP60,* and *OsbZIP50*, are membrane-associated bZIP factors, which fundamentally contribute to ER stress in plant basal immune responses. For example, *Arabidopsis thaliana* gene *bZIP60* encoding an MTF is strongly induced to express by tunicamycin, an ER stress-inducing chemicals (Iwata and Koizumi, 2005), before translocating its protein without TMD into the cell nucleus and upregulating mRNA expression levels of several ER-resident chaperones, sucn as binding protein (BiP) and protein-disulfide isomerases (Iwata and Koizumi, 2005; Lu and Christopher, 2008). In tobacco, expression of *NtbZIP60* was significantly upregulated upon infection with a non-host pathogen *Pseudomonas cichorii*, but not induced by a compatible pathogen *Pseudomonas syringae* pv. *Tabaci* (Tateda et al., 2008). Defense-related plant hormones salicylic acid, induced mRNA splicing of *AtbZIP60* and *OsbZIP50/OsbZIP74* (Hayashi et al., 2012; Lu et al., 2012; Moreno et al., 2012; Parra-Rojas et al., 2015), being the hallmark of IRE1-linking activation of an ER stress regulation defense responses in both Arabidopsis and rice (*Oryza sativa* L.). Abiotic stresses of heat and drought may also lead to splicing of *ZmbZIP60* and *TabZIP60,* (Geng et al., 2018; Li et al., 2018) and *BhbZIP60* mRNAs (Wang et al., 2017), respectively.

In this study, we characterized a gene designated *TabZIP74* from common wheat (*Triticum aestivum* L.), encoding a homologous TF protein of *AtbZIP60* or *OsbZIP74*. The results indicated that the mRNA sequence of *TabZIP74* was spliced in the progress of *Pst* infection and drought stress, which encoded a nucleus-localized factor by frame shift. The spliced mRNA was also detected in stem nodes, roots, and stigmas during normal wheat development. Knocking down the spliced form of *TaZIP74* increased the susceptibility level to stripe rust and decreased drought tolerance. Thus, *TaZIP74* functions as a positive regulator for stripe rust infection and drought tolerance.

## Materials and Methods

### Plant Growth, Biotic Stress, and Chemical Treatments

Wheat near-isogenic lines (NILs) containing the resistant gene *Yr10* (Taichung 29*6/*Yr10*) are resistant to some races of *Pst* in China, while its backcross parent Taichung 29 is highly susceptible (Wan et al., 2004; Chen et al., 2014). We constructed a full-length cDNA library of NIL Taichung 29*6/*Yr10* infected with *Pst* races CYR34 (compatible race) and CYR17 (incompatible race). Wheat seedlings were grown in 8-cm pots and cultivated at 20°C under a 14 h/10 h day/night photoperiod cycle. Seven-day-old seedlings were inoculated with *Pst* races CYR34 and CYR17, or two-week-old wheat seedlings were treated with 5 µg ml^–1^ tunicamycin (TM, ER stress agent) for 4 h. Samples were collected at 0, 6, 12, 24, 36 and 48 h post-inoculation (hpi). To analyze the expression patterns of *TabZIP74* under exogenous plant hormone application and drought stress, 10-day-old seedlings of Taichung 29*6/*Yr10*, cultured in fresh quarter-strength Hoagland solution, were treated with 0.1 mM abscisic acid (ABA) or 20% polyethylene glycol (PEG). Both the leaves and roots of treated plants were sampled at 0, 3, and 6 h post-treatment (hpt). Flag leaves, anthers, stigmas, stem internodes, stem nodes, and roots were sampled at the flowering stage (Feeks 10.5.1). All samples were immediately frozen in liquid nitrogen and stored at –80°C for RNA isolation.

### Gene Expression Analysis

Total RNA of each wheat sample was extracted using TRIZOL reagent according to the manufacturer’s protocol (Invitrogen, USA). The RNA was used to synthesize first-strand cDNA using a TransScript II One-Step gDNA Removal and cDNA Synthesis SuperMix Kit (TransGen Biotech). Reverse transcription (RT)-PCR was performed using TransTaq HiFi PCR SuperMix (TransGen Biotech) and detected by 1.5% agarose gel for gene-spliced assays (Wang et al., 2015). Two pairs of specific primers flanking the splice site were designed to distinguish unspliced (bzipSPassayf1/bzipSPassayr1) and spliced (bzipSPassayf1/bzipSPassayr2) forms of *TabZIP74*. *Ta54227* transcripts of the AAA-superfamily of ATPases (Paolacci et al., 2009) and *NbEF1* were used as controls in the semi-quantitative RT-PCR analyses of unspliced and spliced mRNA forms for expression in wheat or tobacco. Primer sequences are listed in **Table 1**.

**Table 1:** RT-PCR and qRT-PCR primers used for analyzing *TabZIP74* expression in wheat and tobacco.

Name	Sequence (5’-3’)
Ta54227f	CAAATACGCCATCAGGGAGAACATC
Ta54227r	CGCTGCCGAAACCACGAGAC
bzipSPassayf1	ATGAAGTCCAGGGAGAGGAAGA
bzipSPassayr1	GACAGGGAAACCAGCGGCAGAC
bzipSPassayr2	CAGGGTTTCCGAAAGTACGGCA
NbEF1f	TGGTGTCCTCAAGCCTGGTAT
NbEF1r	ACGCTTGAGATCCTTAACCGC

Quantitative RT-PCR (qRT-PCR) was performed on the basis of reported by Wang et al., 2015, GoTaq^®^ qPCR Master Mix (Promega) and ABI7500 Real-Time PCR System (Applied BioSystems) were used. Dissociation curves were generated to ensure specific amplification for each reaction. Each PCR reaction was performed three times. The threshold values (*C* *_T_*) were used to quantify relative gene expression using the comparative threshold (2^-ΔΔ^ *^C^* ^T^) method (Schmittgen and Livak, 2008). *Ta54227* transcripts of the AAA-superfamily of ATPases (Paolacci et al., 2009) were used as a control for the qRT-PCR analyses of the expression level of *TabZIP74* in VIGS plants. Each experiment were performed three replicates. The statistic software SPSS 16.0 (SPSS Inc., USA, http://spss.en.softonic.com/) was used to assy significant differences using one-way ANOVA, taking *P* < 0.05 as significant according to Duncan’s multiple range test.

### Sequence Analysis of *TabZIP74*

Gene sequences were analyzed on the basis of reported by Wang et al., 2015, mainly including DNAMAN software (Lynnon Biosoft, USA) and on line analysis by BLAST and ORF Finder on the NCBI website (http://www.ncbi.nlm.nih.gov/). Multiple sequence alignments were deduced, and a phylogenetic tree generated using the neighbor-joining method with Clustal X version 2.0 (Larkin et al., 2007). The phylogenetic comparison of isolated full-length *TabZIP74*, reported bZIP proteins (Sornaraj et al., 2015) and those derived from GenBank were constructed from the neighbor-joining algorithm using MEGA7 program (Kumar et al., 2016), the bootstrap re-sampling analysis was performed with bootstrap trials = 1000. The sequence of *TabZIP74* was blasted on the Ensembl Plants website (http://plants.ensembl.org/index.html) to get chromosome location of *TabZIP74*.

### Subcellular Localization

To confirm subcellular localization of TabZIP74, fusion-plasmid expression vectors of TabZIP74-eGFP, TabZIP74-eGFPΔC, and eGFP-TabZIP74, containing either a complete or C-terminal truncated sequence of *TabZIP74* cDNA, were constructed (**Figure 3A**). For TabZIP74-eGFP expression vector construction, the encoding region delete the stop codon was amplified by PCR with forward primer sequence TabZIP74subf (5’-TA*GCATCC*ATGGACACCGACCTCGACCT-3’, *BamH* | site in italics) and reverse primer sequence TabZIP74subr (5’-TA*TCTAGA*CTAGCAAGCGGCAGCTGCA-3’, *Xba* | site in italics). For the C-terminal deleted sequence expression vector (TabZIP74-eGFPΔC) construction, the forward primer TabZIP74subf and reverse primer sequence TabZIP74ΔCsubr (5’-TAT*TCTAGA*CGAAAGTACGGCAGACTCCT-3’, *Xba* | site in italics) were used. To construct the eGFP-TabZIP74 expression vector, the encoding region delete the stop codon was amplified by high-fidelity DNA polymerase HIFI Taq (TransGen Biotech) using forward primer eG-bZIP74f (5’-TA*GGATCC*ATGGACACCGACCTCGACCT-3’, *BamH* | site in italics) and reverse primer eG-bZIP74r (5’-AT*TCTAGA*CTAGCAGCGGCAGCTGCA-3’, *Xba* | site in italics). The PCR product was cloned into the binary vectors with eGFP in front or in the back of inserted sequences to produce different fusion vectors. These fusion vector plasmids were introduced into *A. tumefaciens* strain GV3101. Tobacco epidermal cells were infiltrated with *A. tumefaciens* strain GV3101 containing a binary vector encoding GFP-fusion construct for transient expression. 24 h post incubation at 25°C, fluorescence of the GFP images of the transformed tobacco epidermal cells was observed with a confocal microscope (Zeiss LSM 880 Confocal Microscope).

### Transcriptional Activation Analysis in Yeast

To investigate the transcriptional activity of TabZIP74, the complete open reading frame (ORF) and C-terminal deleted cDNA fragment of *TabZIP74* were amplified using the primer combinations (for complete ORF primers: TF1 5’- ATA*GTCGAC*ATGGACACCGACCTCGAC -3’ and TR1 5’- TA*CTGCAG*CTAGCAAGCGGCAGCTGCA -3’; for C-terminal deleted sequence primers: TF1 5’- ATA*GTCGAC*ATGGACACCGACCTCGAC -3’ and primer TR2 5’- TA*CTGCAG*GTTCTGGCGCAGTGCCATGTT -3’, *Pst* I and *Sal* I sites in italics) and fused in the encoding region of GAL4 DNA-binding domain (GAL4-BD) in yeast expression vector pGBKT7, and vectors of pGBKT7-TabZIP74 and pGBKT7-TabZIP74ΔC were constructed for analyzing transcriptional activity of TabZIP74. The empty vector pGBKT7 was used as a negative control. All the vectors were transformed into yeast strain AH109. The different transformants were streaked on medium plates containing SD/Trp- (yeast synthetic drop-out medium supplement without tryptophan) (Clontech, USA) or SD/Trp-/His-/Ade- (yeast synthetic drop-out medium supplement without tryptophan, histidine, or adenine) (Clontech, USA). Incubation at 28°C for 3 d, then evaluated the growth status of the transformants.

### Functional Analysis in Response to *Pst* Infection

Wheat Barley Stripe Mosaic Virus (BSMV)-induced gene silencing assay was conducted as described by Yuan et al. (2011). Specific sequences of wheat *TaPDS* (primer pairs: vTaPDSf, 5’-*AAGGAAGTTTAA*CTGCATAAACGCTTAAAAG-3,’ and vTaPDSr, 5’-*AACCACCACCACCGT*TCTCCAGTTATTTGAG-3’, LIC adapters in italics), *TabZIP74* (primer pairs: vTabZIP74f 5’-*AAGGAAGTTTAA*CCAACCGAAGTCTGGTGGCT-3’ and vTabZIP74r, 5’-*AACCACCACCACCGT*CTAGCAAGCGGCAGCTGCA-3’, ligation-independent cloning (LIC) adapters in italics) were amplified and inserted into vector pCa-γbLIC. For gene function analysis with VIGS, two-week-old common wheat cultivar Fengchan 3 were planted in a growth chamber under a 16 h/8 h day/night photoperiod cycle at 16 ± 2°C. The second leaf surface was inoculated with *Nicotiana benthamiana* leaf sap containing BSMV particles (Mock, BSMV, BSMV-*TabZIP74*, BSMV-*TabZIP74sp*) by gently sliding pinched fingers from the leaf base to the tip. Treated with sterile water as Mock control. The inoculated seedlings were placed in a growth chamber in the dark at 60% humidity, 22 ± 2°C and kept under a 16 h/8 h day/night photoperiod. Nine d after virus inoculation, the phenotypes of wheat seedlings were observed and photographed. Fengchan 3 is highly susceptible to the virulent *Pst* race of CYR32, and its seedlings pre-inoculated with BSMV were successfully infected. Fresh urediniospores of stripe rust race CYR34 were inoculated onto the surface of the third leaves with a paintbrush 14 d after pre-inoculating with the virus. Three independent biological replications were performed for each treatment. The *Pst* infection phenotypes were recorded and photographed from 8 to 16 d post-inoculation (dpi). The latend period and number of uredinia on 6-cm long inoculated leaf fragments was recorded at 16 dpi.

### Functional Analysis in Response to Drought

To examine whether *TabZIP74* is involved in wheat responses to drought stress, the leaf relative water content (RWC) of Mock, BSMV, and BSMV-TabZIP74 plants was determined (Mao et al., 2012). Different virus-pretreated plants were subjected to drought stress by withholding water supply for 10 d, after 10 d rewatering. Development of the treated plants was routinely monitored by record the symptoms of leaf rolling and leaf RWC.

### Measurement of Primary and Lateral Root Lengths

To evaluate the role of *TabZIP74* in the wheat rooting pattern, five-day-old seedlings of Mock, BSMV, and BMSV-TabZIP74 were transferred to fresh quarter-strength Hoagland solution and grown vertically for 20 d at 22°C, under a 16 h/8 h day/night photoperiod. Photographs were recorded with a digital camera, lateral root number in the distal end of primary roots were determined by ImageJ software (http://rsbweb.nih.gov/ij/download.html). The number of lateral roots on 10 plants was calculated, and the mean of lateral roots was used to measure lateral root growth. Three biological replications were performed.

## Results

### Sequence Analysis of Putative *TabZIP74*

**Figure 1:**
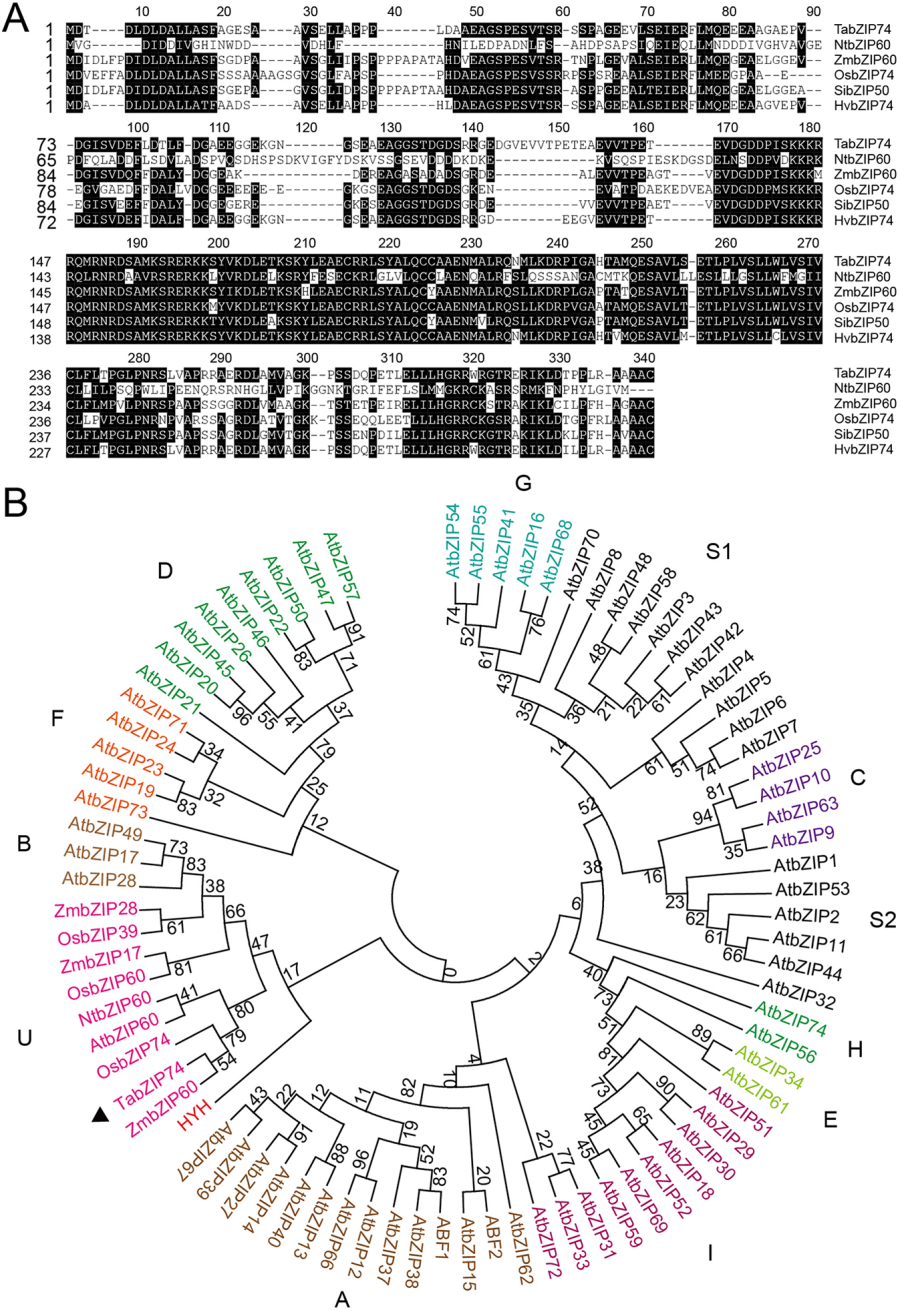
Phylogenetic tree of bZIP transcription factors and alignment of amino acid sequences of TabZIP74. **(A)** Amino acid alignment of TabZIP74 with other bZIP family members of NtbZIP60, OsbZIP74, ZmbZIP60, HvbZIP74, and SibZIP50 from tobacco (*Nicotiana tabacum*, NP_001311663.1), rice (*Oryza sativa*, XP_015641141.1), maize (*Zea mays*, NP_001147256.1), barley (*Hordeum vulgare*, BAJ96708.1), and millet (*Setaria italica*, XP_004965858.1), respectively. The numbers on the left indicate amino acid positions. Identical amino acid residues are shaded black, representing >50% similarity of conserved amino acids. **(B)** Phylogenetic tree of TabZIP74 with selected bZIP TFs from rice (OsbZIPs), Arabidopsis (AtbZIPs), tobacco (NtbZIPs), and maize (ZmbZIPs). Genbank accession numbers of the factors are listed in **Supplementary Table 1**. The subgroups were designated as A, B, C, D, E, F, G, H, S1, S2, and U, according to the analysis results (Jakoby et al., 2002; Zhou et al., 2017).

According to the EST sequence of a differentially expressed bZIP gene in responsing to stripe rust infection, the sequence was cloned from a cDNA library of NIL Taichung 29*6*/Yr10*. The 1,096- base-pair (bp) cDNA clone contains a 909-bp ORF, encoding a putative bZIP74 TF of 302 amino acid residues sharing high identity with MTFs such as *OsbZIP74*, *ZmbZIP60*, *NtbZIP60*, *HvbZIP74,* and *SibZIP50* (**Figure 1A**). Furthermore, genomic sequence blast results indicated *TabZIP74* is located on wheat chromosome 7D.

The phylogenetic tree constructed with TabZIP74 protein and other reported bZIP protein in *Arabidopsis* and rice indicated that different groups of bZIP factors were distinguished and named based on their phylogenetic relationships and functional divergence. The maximum likelihood analysis of bZIP proteins, including TabZIP74, identified 10 bZIP factors from rice, three from maize, one from tobacco and 73 from Arabidopsis in 11 distinct clades (A–I, S, and U), all of which had high bootstrap value support (**Figure 1B**).

The phylogenetic analysis revealed that *TabZIP74* was most homologous to *OsbZIP74* (LOC_Os06g41770) in the rice genome (Correa et al., 2008) and *ZmbZIP60* in maize (*Zea mays* L.) (**Figures 1A, B**). OsbZIP74 also named as bZIP50 (Os06g0622700) in the Rice Annotation Project Database (Wakasa et al., 2011) and is an important ER stress regulator in rice (Lu et al., 2012).

### *TabZIP74* mRNA Splicing in Response to Biotic and Abiotic Factors

**Figure 2:**
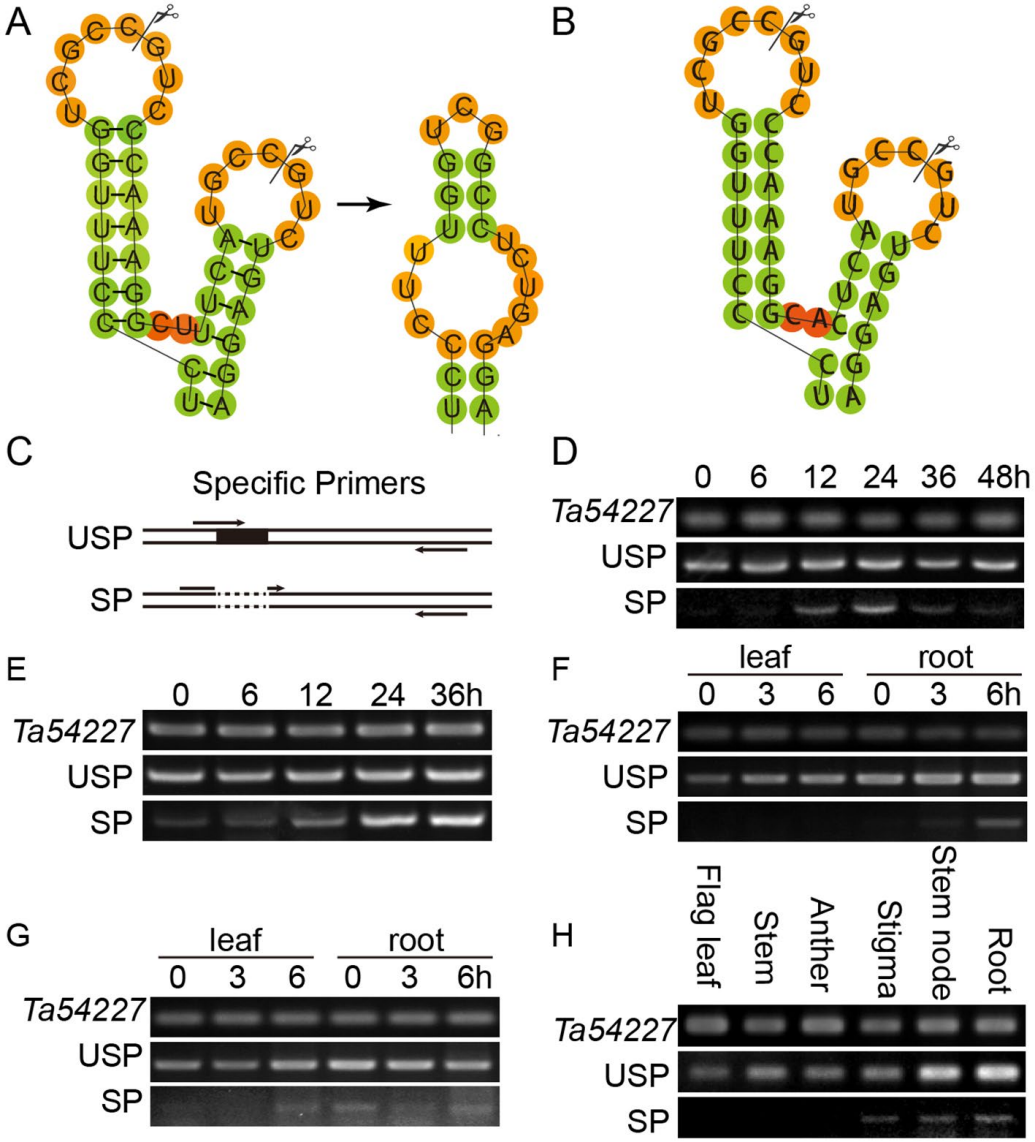
Splicing prediction and molecular assay of *TabZIP74* in response to *Pst* infection, abiotic treatments, and development. **(A, B)** Predicted twin hairpin loop structures of unspliced (USP, left) and spliced (SP, right) forms of *TabZIP74* **(A)** and its homolog of *OsbZIP74* mRNA **(B)**. Each structure contains two stems and two loops. The spliced and predicted cleavage sites are highlighted with scissors. Schematic representation of the USP and SP primers **(C)**, Time-course experiments of the *TabZIP74* splicing in response to infection by *Pst* with an avirulent race CYR17 **(D)** and a virulent race CYR32 **(E)**. Splicing test in wheat seedling leaves and roots under PEG-induced drought stress **(F)** and ABA **(G)**. **(H)** Splicing test in flag leaves, stems, anthers, stigmas, stem nodes, and roots of adult wheat plants.

TabZIP74 has a high level of identity to OsbZIP74 with regard to amino acid sequences. Interestingly, the mRNA sequence of *TabZIP74* can form two kissing hairpin loops through an RNA secondary structure prediction program Centroidfold (http://rtools.cbrc.jp/centroidfold/) (Hamada et al., 2009), and potentially produce its unspliced (*TabZIP74-USP*) and spliced (*TabZIP74-SP*) forms. The spacer between two hairpin loops in *TabZIP74* comprises only two nucleotides, the same as *OsbZIP74* (**Figures 2A, B**).

Speicific primers flanking the predicted *TabZIP74* splice sites was designed and used for RT-PCR analysis to assay the splicing of *TabZIP74 *(**Figure 2C**). When wheat seedling leaves were treated with 5 µg ml^–1^ tunicamycin (TM) for 4 h, one normal cDNA band and another smaller one were detected with RT-PCR in agarose gel electrophoresis. The sequencing result showed that a 20 bp fragment was spliced from the original *TabZIP74* mRNA molecule in response to TM treatment (**Figure 2A**). The specific primers were also used to discriminate the unspliced (USP) and spliced (SP) forms of *TabZIP74* after infection with *Pst*. When wheat seedlings were inoculated with two *Pst* races (CYR17, avirulent; CYR34, virulent), both induced *TabZIP74* mRNA molecules for splicing (**Figures 2D, E**). In contrast, the abiotic factor PEG triggered *TabZIP74* to be spliced in the roots of treated wheat seedlings (**Figure 2F**), while ABA induced splicing in both leaves and roots of stressed seedlings (**Figure 2G**). *TabZIP74* mRNA splicing also occurred in stigmas, stem nodes, and roots of adult wheat plants (**Figure 2H**), but not in unstressed seedling leaves (**Figures 2 D–F, H**).

### Subcellular Localization of TabZIP74

**Figure 3:**
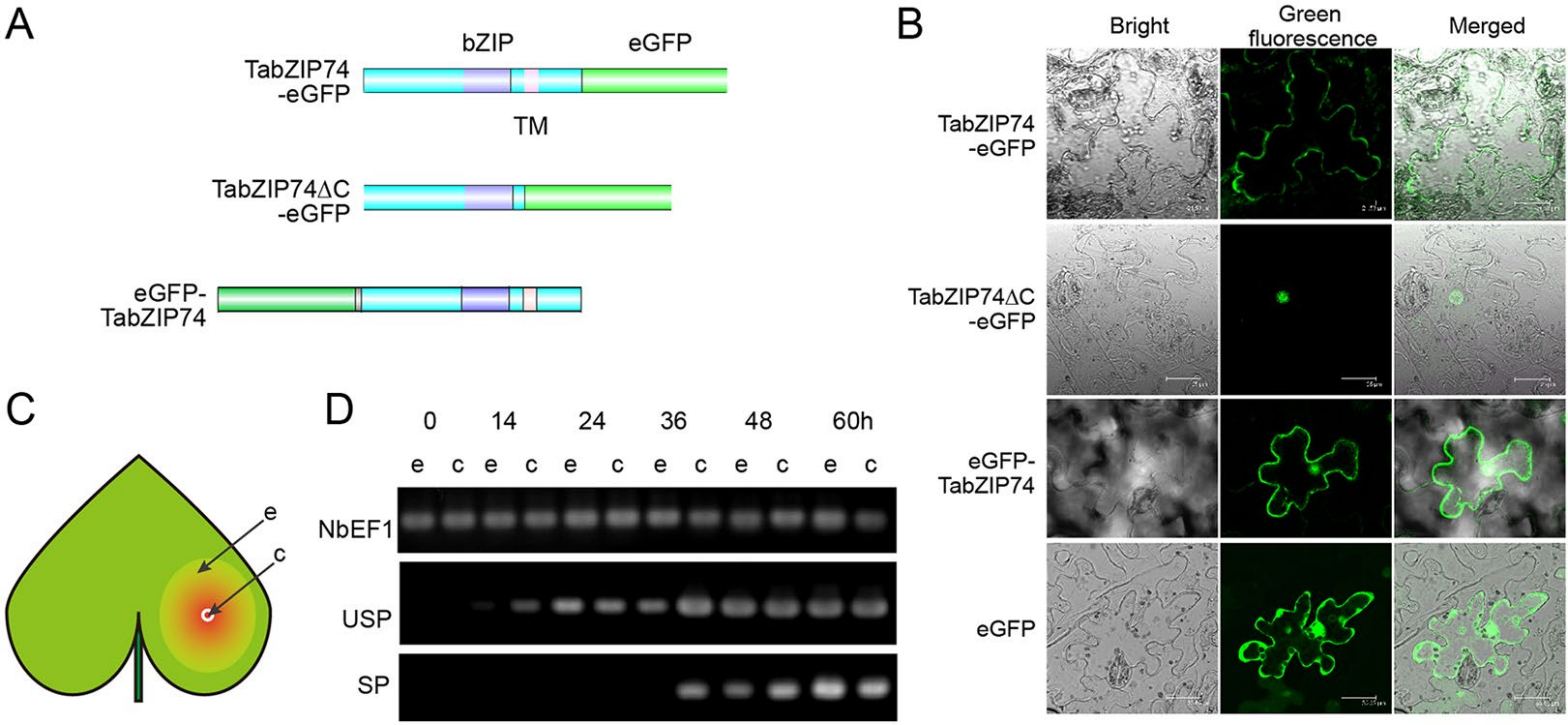
Subcellular localization of TabZIP74 and its mRNA splicing in tobacco leaf epidermal cells. **(A)** Plasmids encoding fusion proteins of eGFP with TabZIP74 were constructed, including two structures of full-length TabZIP74 fused to the N- or C-terminal of eGFP (top and bottom) and truncated TabZIP74(ΔC) (middle) fused to the N-terminal of eGFP protein. **(B)** Subcellular localization of TabZIP74 protein. **(C)** Schematic diagram of tobacco leaf infiltration. ‘c’ and ‘e’ represent the ‘center’ and the ‘edge’ of infiltrating area inoculated with *A. tumefaciens* cells containing GFP-fusion vectors, respectively. **(D)** mRNA splicing assay of *TabZIP74* induced by wounding in tobacco leaf cells. USP and SP, unspliced, and spliced mRNA forms of *TabZIP74*. *NbEF1* was used as control genes in the tobacco leaf RT-PCR analyses.

To investigate the subcellular localization of TabZIP74 in plant cells, different types of fusion protein with eGFP were constructed and these fusion vectors were transiently expressed in *Nicotiana benthamiana* leaf epidermal cells infiltrated with *Agrobacterium tumefaciens* cells of strain GV3101, containing a vector of TabZIP74-eGFP, TabZIP74-eGFPΔC or eGFP-TabZIP74 (**Figure 3A**). Confocal microscopic examination showed that the TabZIP74-GFP protein bound only to the plasma membrane and the TabZIP74-ΔC-GFP protein bound to the nuclei of tobacco epidermal cells, whereas eGFP-TabZIP74 was localized in both the plasma membrane and nucleus (**Figure 3B**). These results suggest that TabZIP74 is a membrane-bound and nucleus-localized protein.

The mRNA levels of USP and SP forms of *TabZIP74* in tobacco leaf epidermal cells infiltrated with the strain GV3101 with a fusion vector of *eGFP-TabZIP74* was assayed by semi-RT-PCR. Fourteen hours after infiltrating, USP-type mRNA molecules of *TabZIP74* were detected at the inoculation site center with a high expression level and at the edge of infiltrated leaves with a relatively lower level. The SP-type molecules of *TabZIP74* were not detected at the inoculation site center until 36 h after infiltration, and no SP forms were found at the edge of the infiltrated area; both types of *TabZIP74* mRNA were not detected at the inoculation site center or the edge of infiltrated leaves until 14 h after infiltration (**Figures 3C, D**). Therefore, wounding by infiltration possibly induces *TabZIP74* mRNA splicing in tobacco cells, and the proteins encoded by the confusion gene *eGFP-TabZIP74* were bound to the tobacco epidermal cell membrane and accumulated in the nuclei, while those encoded by *TabZIP74-eGFP* were only bound to the plasma membrane (**Figure 3B**).

### Transcription Activity of TabZIP74

**Figure 4:**
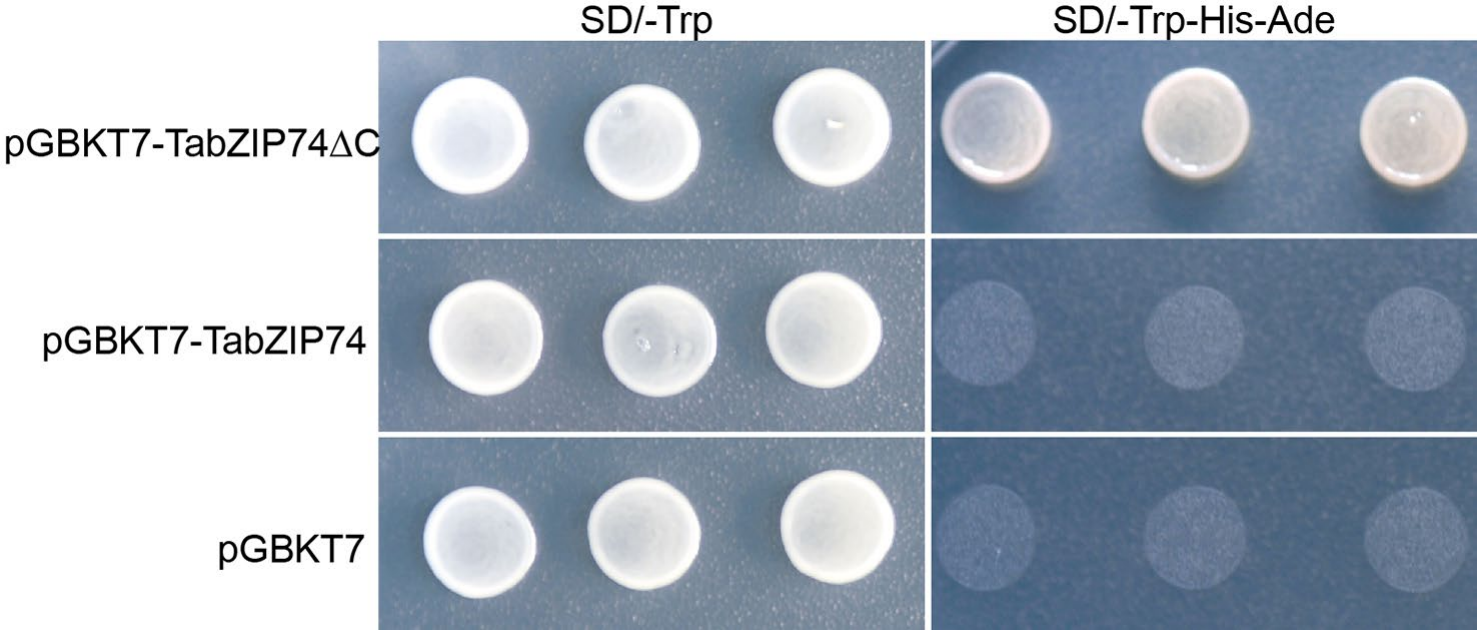
Transcription activity assay of TaZIP74 protein. The full-length encoding region and truncated cDNA fragment of *TabZIP74* were fused with the GAL4 DNA-binding domain in vector pGBKT7. The plasmid containing the fusion gene and the empty vector control pGBKT7 were introduced into yeast cells of strain AH109. The yeast transformants were plated and incubated for 2 d at 28°C on SD/Trp− plate and SD/Trp−/His−/Ade− plate.

The full-length ORF and its truncated cDNA fragment without TMD (*TabZIP74ΔC*) were fused into the GAL4-DB in the vector pGBKT7, and the constructs were transformed into yeast strain AH109 cells to assay TabZIP74 transcriptional activity. The yeast transformant cells containing the fusion plasmids of full-length cDNA of *TabZIP74*, *TabZIP74ΔC,* and the vector control pGBKT7 grew well on selection SD medium plate without tryptophan (Trp−) (**Figure 4**). However, only the yeast cells encoding the fusion protein of TabZIP74ΔC grew well on the selection medium without tryptophan, histidine, and adenine (Trp−/His−/Ade−) (**Figure 4**), indicating that TabZIP74ΔC had transcriptional activity in yeast cells.

### *TabZIP74* Knockdown Plants Increased Susceptibility to *Pst*

**Figure 5:**
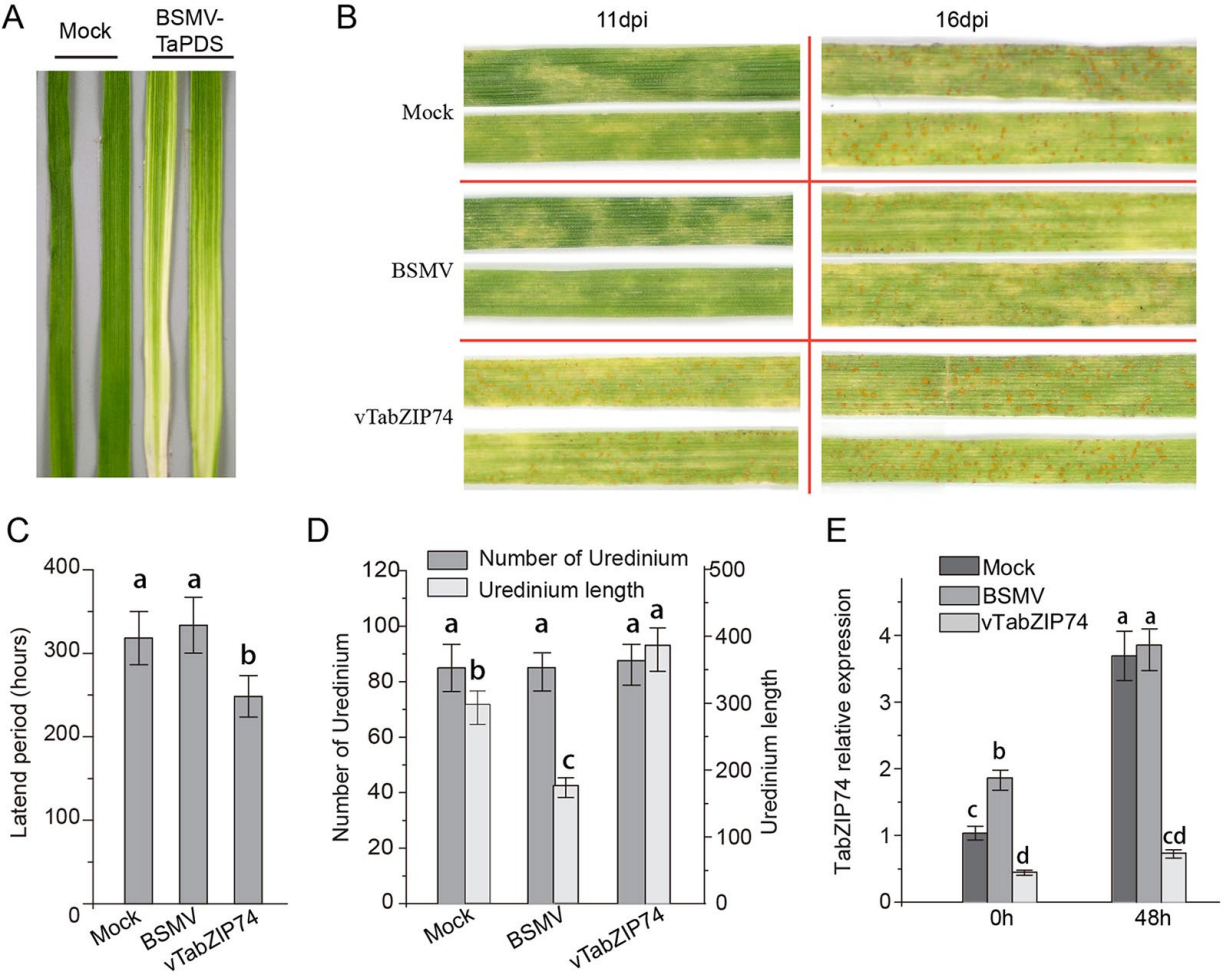
Functional characterization of *TabZIP74* in response to *Pst* infection using the BSMV-VIGS system. **(A)** Third leaves of wheat seedlings of cultivar Fengchan 3 pre-inoculated with positive control vector (BSMV-*TaPDS*) at 14 d post-virus treatment (dpi). **(B)** Phenotypes for the third leaves inoculated with *Pst* race CYR32 were observed at 11 dpi and 16 dpi, respectively; the second leaves of these seedlings were pre-inoculated with water (Mock), empty BSMV vector and silencing vector of BSMV-*vTabZIP74*. **(C)** *Pst* latent infection period of different treatments. **(D)** Statistics of uredinium number and length on third leaves inoculated with *Pst*. Representative experiments were replicated three times (*n* = 3), at least 60 uredinia on 6-cm leaf segments from Mock, BSMV vector and BSMV-*vTabZIP74* treated seedlings were counted in each replicate. Error bars indicate SD, letters indicate significant differences between Mock, BSMV, and BSMV-*TabZIP74* samples determined by one-way ANOVA, taking *P* < 0.05 level according to Duncan’s multiple range tests. **(E)** Silencing efficiency of *TabZIP74* expression in knockdown leaves was determined by qRT-PCR; third leaves were sampled at 0 and 48 h after inoculation with BSMV virus. All experiments of *TabZIP74* functions for *Pst* infection were replicated five times, and the results of three representatives were selected.

To identify the regulatory roles of *TabZIP74* in the wheat response to *Pst* infection, the specific C-terminal fragment of *TabZIP74* was used to construct the BSMV-*TabZIP74* fusion vector to silence *TabZIP74* expression in wheat seedlings. Mild chlorotic mosaic symptoms were observed in the BSMV-inoculated plants 10 d post virus inoculated, and there was no obvious defects in further seedling leaf growth. Typical photobleaching was observed on the leaves of wheat seedlings pre-inoculated with BSMV-*TaPDS* (a specific fragment of wheat phytoene desaturase gene *PDS*) 14 d after virus inoculation, indicating the feasibility of the gene knockdown system applied in this study (**Figure 5A**). Interestingly, 11 dpi with the virulent *Pst* race of CYR 32, uredinium pustules erupted on the *TabZIP74*-knocked down seedling leaves of variety Fengchan 3, while no uredinia were visible on the Mock or vector control plants (**Figure 5B**). As a result, the latent period of *Pst* infection in silenced plants was significantly shorter than in the BSMV vector control and Mock seedlings (**Figure 5C**). Sixteen d post-inoculation, most *Pst* uredinia had matured and erupted from the seedling leaf surface (**Figure 4B**). There were no significant differences in the number of *Pst* uredinia on Mock, BSMV control and *TabZIP74*-knocked down seedling leaves. However, the *TabZIP74*-knocked down seedling leaves had longer uredinia than the Mock and BSMV control seedlings (**Figure 5D**). The expression level of *TabZIP74* before *Pst* inoculation was ranked BSMV > Mock > *TabZIP74*-knocked down seedlings. At 48 hpi, no differences in expression level were detected between BSMV and Mock treatments, but the *TabZIP74* expression level remained relatively low in its knockdown seedlings (**Figure 5E**).

### *TabZIP74* Knockdown Plants Decreased Drought Tolerance and Lateral Roots

**Figure 6:**
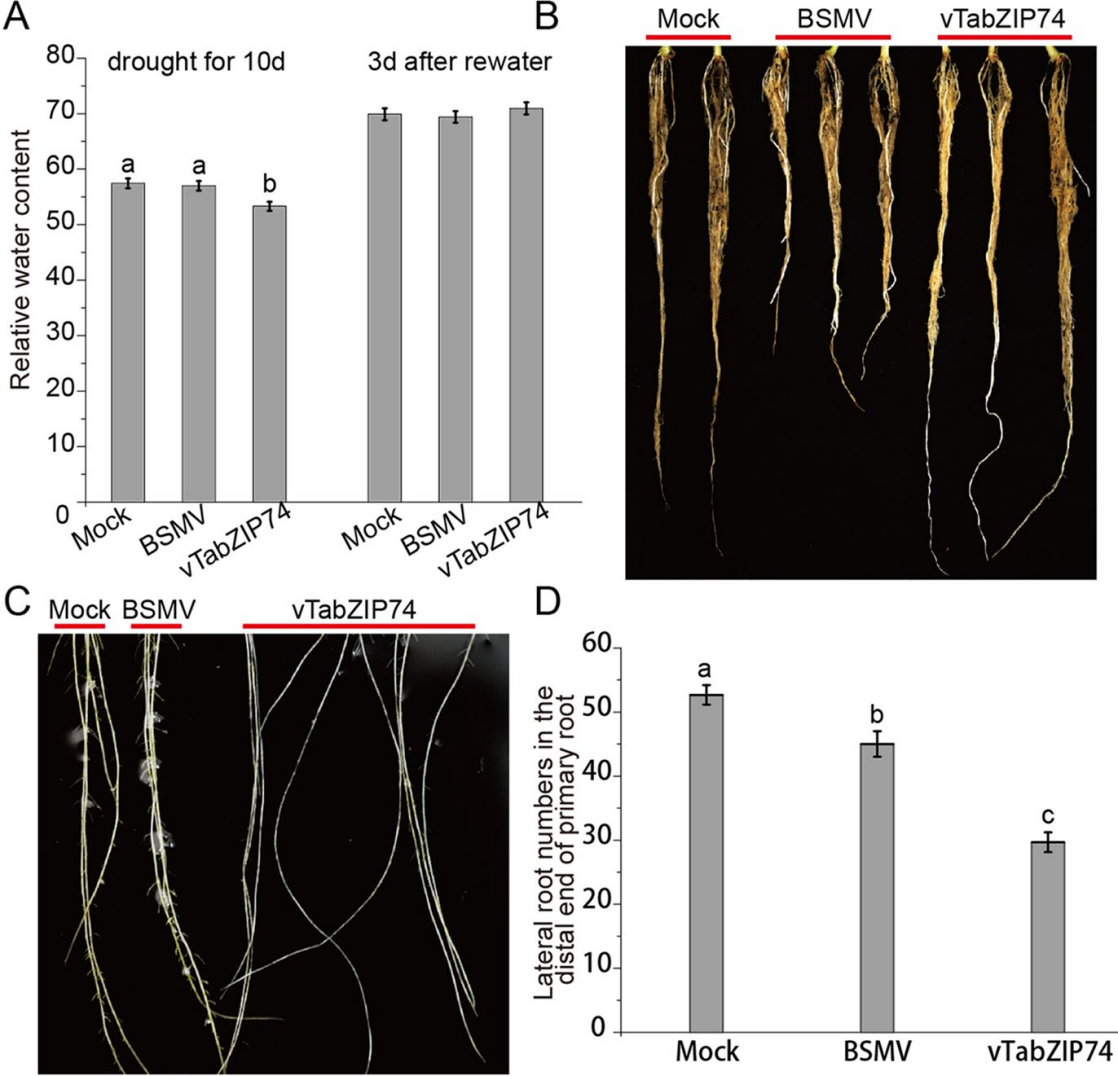
The effects of *TabZIP74* silencing on wheat seedling drought tolerance and root development. **(A)** RWC of plants exposed to drought for 10 d and rewatered 3 d later. The experiments were performed in triplicate. Each value is the mean ± standard deviation of three independent measurements (SD, n = 3). Different letters indicate significant differences by one-way ANOVA, taking *P* < 0.05 level according to Duncan’s multiple range test for comparisons between treatment times. **(B)** Morphological differences in primary root development between Mock, BSMV, and BSMV-*vTabZIP74* plants. **(C)** Morphological differences in lateral root development between Mock, BSMV, and BSMV-*vTabZIP74* plants cultured in quarter-strength Hoagland solution for 20 d after sowing. **(D)** Lateral root numbers in the distal end of the primary root of Mock, BSMV, and BSMV-*vTabZIP74* plants cultured in 10-cm pots for 20 d after sowing.

Leaf RWC is an important indicator of plant drought response, reflecting the water balance in leaf tissues. Wheat seedlings withholding water for 10 d to drought stress, and then rewatering for 3 d, and the RWC of seedling leaves was assayed at different drought stages. There was no notable phenotypical difference in leaf rolling or wilting between treatments, but the drought-stressed plants pre-silenced by BSMV-*vTabZIP74* had lower RWC than Mock and BSMV control plants; 3 d after rewatering, RWC did not differ between treatments (**Figure 6A**). There were clear differences in primary root length of treated plants, namely, *TabZIP74* > Mock > BSMV (**Figure 6B**). Plants knocked down with *TabZIP74* developed longer primary roots but had less drought tolerance than Mock- and BSMV-treated plants. Furthermore, 9 d post-virus inoculation, *TabZIP74*-knockdown plants had significantly fewer lateral roots than BSMV-infected and Mock plants (**Figures 6C, D**).

## Discussion

Upon pathogen recognition, plants responsed rapid and complex immune responses. One type of plant defense response is the programmed burst in transcription and translation of pathogenesis-related proteins, most of which rely on ER processing (Korner et al., 2015). Two plant ER stress sensors, bZIP28 and IRE1, are involved in ER stress-induced signaling (Yoshida et al., 2001; Deng et al., 2011), but only IRE1 has been shown to operate in plant immune responses (Moreno et al., 2012). Plant bZIP TFs, such as AtbZIP60 and OsbZIP50/OsbZIP74, are involved in Regulated IRE1-Dependent Splicing (RIDS) in response to ER stress (Deng et al., 2011; Hayashi et al., 2012).

### mRNA Splicing of *TabZIP74* Was Initiated by *Pst* Infection and Wounding

TabZIP74 encodes an ORF consisting of 302 amino acids, beside bZIP DNA-binding domain there was a TMD in C terminal. The ER stress lead TabZIP74 mRNA splicing, and the spliced gene encode noval protein without TMD. The phylogenetic tree indicated that TabZIP74 belong to the subgroup U, most of its members have been characterized to regulate UPR. For example, *ZmbZIP60* mRNA is spliced in maize in response to ER stress (Li et al., 2012), OsbZIP74 is an important ER stress regulator in rice (Lu et al., 2012), OsbZIP39 regulates the ER stress response in rice (Takahashi et al., 2012), and NtbZIP60 is an ER-localized transcription factor in tobacco (Tateda et al., 2008; Xu et al., 2013). Therefore, TabZIP74 may be involved in ER stress responses.

IRE1 has been proved as a dual protein kinase/RNase. The predicted structure of the IRE1 splicing site was based on two ‘kissing’ hairpin loops with conserved bases, and its predicted cleavage sites located close to the ribonuclease catalytic sites in its cytosolic domain (Lee et al., 2008). *TabZIP74* shares high similarity with *OsbZIP74* in the nucleotide sequence, neither of which had a higher sequence identity than the bZIP TF of yeast *HAC1* or mammalian *XBP1* at nucleotide or protein levels in response to ER stress (Kawahara et al., 1998; Yoshida et al., 2001). *TabZIP74* and *OsbZIP60* mRNAs, being like *HAC1* and *XBP1* mRNAs, can fold and form an IRE1 recognition site with two stem loops, each containing the bases remarkably conserved from yeast to mammals at three positions.

The splicing of *TabZIP74* mRNA in wheat leaves or roots was evident from RT-PCR and sequencing after ER stress, *Pst* infection, or drought and ABA treatments. The mRNA splicing of *TabZIP74* was also detected in tobacco leaves infiltrated with *A. tumefaciens* cells containing the *GFP-TabZIP74* fusion vector. Consequently, the *TabZIP74* mRNA sequence in infiltrated tobacco leaves may have been spliced by IRE1 under ER stress triggered by wounding.

### TabZIP74 mRNA Splicing Produces the Active Form of Transcription Factor bZIP74

The full length of TabZIP74 was located on the membrane, and the truncated form of TaZIP74 can enter the nucleus (**Figure 3A**); OsbZIP74, AtbZIP60, ZmbZIP60, and NtbZIP60 showed similar results. In this research, we fused eGFP in the N terminus and found the splicing of TabZIP74 lead to new proteins entering the nucleus. The IRE1 splicing in mammals produces the active form of the transcription factor by adding the transcriptional activation domain in the newly formed longer C-terminus (Lu et al., 2012). In this research, the truncated but not full length of TabZIP74 acts as a transcriptional activator in yeast; similar results were observed in NtbZIP60. However, the TMD in OsbZIP74 and AtbZIP74 did not affect activation activity in yeast. So, the splicing of TabZIP74 produces the active form of transcription factor bZIP74.

### BSMV Infection Enhanced the Expression Level of *TabZIP74*

Plant-infecting viruses can activate the ER stress signaling mechanism. Upon infection, viruses hijack cellular machinery to replicate their genomes and translate viral proteins (Korner et al., 2015). In Arabidopsis, when leaves were infected with potyvirus *Turnip mosaic virus* (Gaguancela et al., 2016) the spliced bZIP60 mRNA were accumulated. ER stress marker genes were also induced in *N. benthamiana* infected with potexvirus *Potato virus X* (PVX) and fijivirus *Rice black-streaked dwarf virus* (Ye et al., 2011; Sun et al., 2013). And it has proved that ER stress activation acts as a positive regulator of virus replication (Korner et al., 2015).

Our results showed that the infection of BSMV induced a higher expression level of *TabZIP74* than Mock or BSMV-*TabZIP74* (**Figure 4F**). Moreover, the stripe rust disease severity of BSMV-treated seedlings was lower than Mock and BSMV-*TabZIP74* seedlings (**Figure 4E**). As such, the wheat seedlings pre-infected with BSMV may reduce the disease severity of stripe rust.

### *TabZIP74* Involved in Wheat Drought Tolerance and Development

In plants, UPR is provoked by a heavy demand in anther tapetal cells to synthesize and secrete pollen coat materials (Deng et al., 2016). Our study is also showed that UPR contributes to normal plant development. Under normal development conditions, low levels of the spliced form of *TabZIP74* were detected in wheat stem nodes, roots, and stigmas. It is interesting to note that *TabZIP74* knockdown seedlings developed fewer lateral roots, while other studies have found that IRE1a and IRE1b support root growth in ways other than the splicing of *bZIP60* mRNA (Deng et al., 2016). The growth of single or double *bZIP60* mutants in combination with either *bZIP17* or *bZIP28* appear to be normal in unstressed conditions (Kim et al., 2018; Bao et al., 2019). Biotic stress, ER stress-inducing agent, ABA treatment, and drought could induce *TabZIP74* mRNA splicing. However, in rice, the ER stress-inducing agent and SA treatment induced the splicing of *OsbZIP74*, the homolog of *TabZIP74*, whereas stress hormone ABA and drought did not. So differences in biological function may exist between TabZIP74 and OsbZIP74.

The wheat root system includes the primary root, crown, and lateral roots, and root hairs. When *TabZIP74* expression was silenced with VIGS, the wheat seedlings developed longer primary roots, but with significantly fewer lateral roots. This may be the reason why the *TabZIP74* knockdown plants showed less drought tolerance (**Figure 5**). Besides, *TabZIP74* knockdown plants also increased susceptibility to *Pst* infection. Water deficiency, pathogen infection, or stress-induced agent ABA usually cause the disorder of protein synthesis, degradation, and folding. In the endoplasmic reticulum, it is termed ER stress. Thus, it is particularly important to maintain protein stability in plant cells. ABA plays a major role in abiotic stress signaling, in particular in drought and salinity stress responses. ABA also has a pivotal role in the regulation of the plant immune signaling network (Pieterse et al., 2012). In Arabidopsis, ABA signaling antagonizes plant immunity by suppressing SA-dependent defenses. *Boea hygrometrica* bZIP transcription factor, BhbZIP60, is a splicing-activated ER stress regulator involved in drought tolerance. So, we propose that the splicing form of *TabZIP74* is involved in the ABA pathway to respond to abiotic and biotic stresses.

In brief, *TabZIP74* encodes a membrane-associated bZIP-type transcription factor. Based on the results presented in this study, we conclude that *TabZIP74* might positively regulate wheat defenses against *Pst* and drought stress tolerance and is necessary for lateral root development.

## Data Availability Statement

The raw data supporting the conclusions of this manuscript will be made available by the authors, without undue reservation, to any qualified researcher.

## Author Contributions

RL and WC designed the experiment. RL and FW wrote the manuscript. FW, YL, PW, JF, and SX performed the experiments and analyzed the data.

## Funding

The study was financially supported by the National Key Research and Development Program of China (projects 2018YFD0200501, 2018YFD0200401) and National Natural Science Foundation of China (31871949).

## Conflict of Interest

The authors declare that the research was conducted in the absence of any commercial or financial relationships that could be construed as a potential conflict of interest.

## Supplementary Material

Supplementary Material includes one table listing Locus ID of bZIP genes.
